# Avian Flavivirus Enters BHK-21 Cells by a Low pH-Dependent Endosomal Pathway

**DOI:** 10.3390/v11121112

**Published:** 2019-11-30

**Authors:** Abdul Sattar Baloch, Chunchun Liu, Xiaodong Liang, Yayun Liu, Jing Chen, Ruibing Cao, Bin Zhou

**Affiliations:** MOE Joint International Research Laboratory of Animal Health and Food Safety, College of Veterinary Medicine, Nanjing Agricultural University, Nanjing 210095, China; balochsattar@ymail.com (A.S.B.); crb@njau.edu.cn (R.C.)

**Keywords:** Duck Tembusu virus, endocytosis, low pH, clathrin, proteasome

## Abstract

Duck Tembusu virus (DTMUV), a pathogenic member of the *Flavivirus* family, was first discovered in the coastal provinces of South-Eastern China in 2010. Many previous reports have clearly shown that some *Flaviviruses* utilize several endocytic pathways to enter the host cells, however, the detailed mechanism of DTMUV entry into BHK-21 cells, which is usually employed to produce commercial veterinary vaccines for DTMUV, as well as of other *Flaviviruses* by serial passages, is still unknown. In this study, DTMUV entry into BHK-21 cells was found to be inhibited by noncytotoxic concentrations of the agents chloroquine, NH_4_Cl, and Bafilomycin A1, which blocked the acidification of the endosomes. Inactivation of virions by acid pretreatment is a hallmark of viruses that utilize a low-pH-mediated entry pathway. Exposure of DTMUV virions to pH 5.0 in the absence of host cell membranes decreased entry into cells by 65%. Furthermore, DTMUV infection was significantly decreased by chlorpromazine treatment, or by knockdown of the clathrin heavy chain (CHC) through RNA interference, which suggested that DTMUV entry depends on clathrin. Taken together, these findings highlight that a low endosomal pH is an important route of entry for DTMUV.

## 1. Introduction

Duck Tembusu virus (DTMUV) is a mosquito-borne *Flavivirus*, and a member of the *Flaviviridae* family. The virus is comprised of a single-stranded and positive-sense RNA genome which encodes three structural proteins (envelope, E; membrane precursor, PrM; and capsid C) and seven nonstructural proteins (NS1, NS2A, NS3, NS4A, NS4B, and NS5) and has an open reading frame (ORF). DTMUV envelope protein (E) is the main surface protein and plays a vital role in receptor binding and successive fusion events between the virus and host membranes [1,2,3]. DTMUV infection in ducks is represented by a variety of signs, such as decline in egg production, internal bleeding, diarrhea, acute anorexia, and paralysis. The infection rate is up to 90% and the consequent mortality rate is as high as 30% [4]. DTMUV has become a prevalent contagious disease in ducks, leading to severe economic losses in the duck industry in China [5]. In addition, DTMUV manifests with a wide range of hosts including other avian species such as, chickens, geese, and sparrows; and, like other *Flaviviruses*, DTMUV has a zoonotic nature. Recent studies have revealed that humans can be infected with DTMUV [6,7,8].

Great progress has been made in the study of the etiology, molecular biology, and immunological characteristics of DTMUV [9], and the use of vaccines has decreased the incidence of DTMUV [10]. Factors such as immune failure and immunosuppression often lead to an epidemic of DTMUV [11], and there are no effective antiviral drugs available in china. Moreover, the issue of the pathogenesis and antiviral research on DTMUV has become both the focus of and difficulty in current research. The binding of the virus to the corresponding receptors on the cell surface is the initiating link in the life cycle of the virus, and it is also one of the decisive factors affecting the host range, tissue tropism, and pathogenicity of the virus [12]. Viral entry has thus become an attractive target for antiviral therapies and antiviral drug design [13]. Several enveloped *Flaviviruses* utilize different endocytic methods penetrating into host cells [14]. *Flaviviruses* such as Dengue, West Nile, and JEV enter cells via receptor-mediated endocytosis and low pH pathways [15,16]. Viral entry mechanisms are extensively characterized by interaction between the virus and host cell receptors [14]. *Flaviviruses* access cells through receptor-mediated endocytosis, after endocytosis the virion is deposited into the endosomes, whereby a moderately acidic pH is required for productive entry [17,18,19]. However, the mechanism of DTMUV entry is unknown. Therefore, this is the first report on DTMUV entering BHK-21 cells through a low pH. In this study, we evaluated the mechanism of DTMUV entry into BHK-21 cells. We used lysosomotropic agents (chloroquine and NH4Cl) and Bafilomycin A1, vacuolar ATPase siRNA, and proteasome inhibitor MG-132, to examine the DTMUV internalization mechanism. We further confirmed that DTMUV entry depends on clathrin. Overall, the findings indicated that DTMUV entered BHK-21 cells by low pH-dependent and proteasome mediated endocytosis which requiring clathrin.

## 2. Material and Methods

### 2.1. Cells and Virus 

Dulbecco’s modified essential medium (DMEM, GIBCO, Invitrogen, Carlsbad, CA, USA) supplemented with 10% fetal bovine serum (FBS, GIBCO, Grand island, NY, USA), 100 μg/mL streptomycin, and 100 IU/mL penicillin (GIBCO, Grand island, NY, USA) was used for the baby hamster kidney 21 (BHK-21, ATCC CCL-10) cell culture, at 37 °C in a 5% CO2 incubator. The Duck Tembusu virus DTMUV strain XZ-2012, provided by Prof. Ruibing Cao, Nanjing Agricultural University (Genbank accession number: KM188953) was cultured in BHK-21 cells supplemented with 2% FBS. Two to three days later, following freezing and thawing three times, medium was harvested, centrifuged at 1000× *g* for 10 min, and filtered with 0.22 μM Nest to remove the cells and cellular debris. Virus aliquots were kept at −80 °C.

### 2.2. Cell Infection and Drug Treatments

BHK-21 cells were seeded in six-well plates for one to three days until they reached 70% confluence and were then treated with the indicated concentrations of chloroquine (Sigma), NH4Cl (Sigma), chlorpromazine CPZ (Sigma, Saint Loius, USA), Bafilomycin A1 (Baf A1; Cayman, Michigan USA), and MG-132 (MCE, NJ, USA), for 1 h at 37 °C before or during viral infection, in order to test the effects of various drugs on DTMUV infection. Adsorption and internalization of DTMU Virus was achieved by infecting the cells at an MOI of 1 at 4 °C for 1 h in the presence of the drug, and then shifting them to an incubator at 37 °C for binding and entry for 0 and 1 h, respectively. After that, cell lysing was achieved by three cycles of freeze-thaw. A replication assay was undertaken by infecting the cells at an MOI of 0.1 in the presence of the drug for 1 h, before further incubation in the absence of the compound. The same experimental protocol was performed at 24 h post-infection (hpi) as described above. Briefly, a time of addition assay was also undertaken. Cells were infected with an MOI of 0.5 for 1 h, and lysosomotropic agents were added at 0, 1, 3, and 6 h. After 9 hpi RT-qPCR and Confocal microscopy were performed.

### 2.3. RNA Extraction and RT-qPCR

The total RNA in infected cells was extracted, using TRIzol reagent (TaKaRa Bio, Kyoto, Japan). Then, the RNA was reverse transcribed into cDNA. The level of DTMUV was determined by RT-qPCR using Eva green 2× qPCR master mix (Abm, Canada) in at 7300 real-time PCR system (Applied Biosystems, Foster City, CA, USA). Two pairs of specific primers were designed to amplify the DTMUV E gene and the cellular β- actin in the conserved region by utilizing DNAMan software. These were, respectively, (DTMUV-E-F: 5′-TGTCTTATGCAGGTACCGATG-3′, DTMUV-E-R: 5′-CGTATGGGTTGACTGTTATCA-3′; actin-F:5′-CTCCATCATGAAGTGCGACGT-3′, actin-R:GTGATCTCCTTCTGCATCCTGTC-3′). The data analysis is presented as the 2-ΔΔCT value from quadruplicate samples [20].

### 2.4. Cell Viability Assay 

A CellTiter 96 Aqueous one solution cell proliferation assay kit (Promega) was used to analyze the cytotoxic effect of the drugs on BHK-21 cells as per the manufacturer’s protocol. Noncytotoxicity was examined in cells treated with the indicated concentrations of the drugs.

### 2.5. Confocal Microscopy 

BHK-21 cells were seeded on glass coverslips in six-well plates. When they reached 70% confluence, the cells were infected with DTMUV at an MOI of 0.5 for 1 h at 37 °C. After 24 h of infection with DTMUV, the fixation of the monolayer of the cells was achieved with 4% paraformaldehyde for 15 min. They were permeabilized with 0.1% Triton X-100, and then stained with mouse anti-DTMUV E monoclonal antibody. The coverslips were mounted and the cells were observed using confocal microscopy Nikon Eclipse Ti, Japan.

### 2.6. siRNA Transfections

Small interference RNAs siV-ATPase (sc-36787), siCHC Clathrin heavy chain (sc-35067), and negative-control siRNA (sc-37007) all were purchased from Santa Cruz Biotechnology, Inc. Before siRNA transfection, the BHK-21 cells were cultured in six-well plates at 2.5 × 10^5^ cells/well and then transfected with 100 nM siRNAs using lipofectamine 3000 (invitrogen). The protocol was performed as per the manufacturer’s directions. After 24 h post transfection, the cells were infected with DTMUV at an MOI of 0.1, and the efficiency of the siRNAs was analyzed by quantified RT-qPCR and Western blotting, as described above. For the internal control, β-actin was used.

### 2.7. Western Blotting

Before lysing of the cells with lysis buffer RIPA for 15 min, the cells were washed three times with PBS. Centrifugation at 12,000× *g* for 10 min was undertaken to clarify the protein lysate. SDS-PAGE was performed to separate the proteins in the lysates, then they were transferred to nitrocellulose membranes, and probed with the indicated antibodies. Rabbit anti-clathrin (P1663) CST and mouse anti-V-ATPase B 1/2 antibody (sc-271832) were obtained from Santa Cruz Biotechnology Inc. DTMUV E monoclonal antibody was a kind gift from Prof. Renyong Jia (Sichuan Agricultural University). β-actin served as an internal loading control.

### 2.8. Statistical Analysis

GraphPad Prism 7 (GraphPad Software Inc, La Jolla, CA) was used for the statistical analyses. The data were represented as means ± standard deviations (SD), as indicated in the figure legends. A Student’s *t*-test was applied to the data to for compare the pairs of treated or untreated groups. 

## 3. Results

### 3.1. DTMUV Entry is pH Dependent

**Figure 1:**
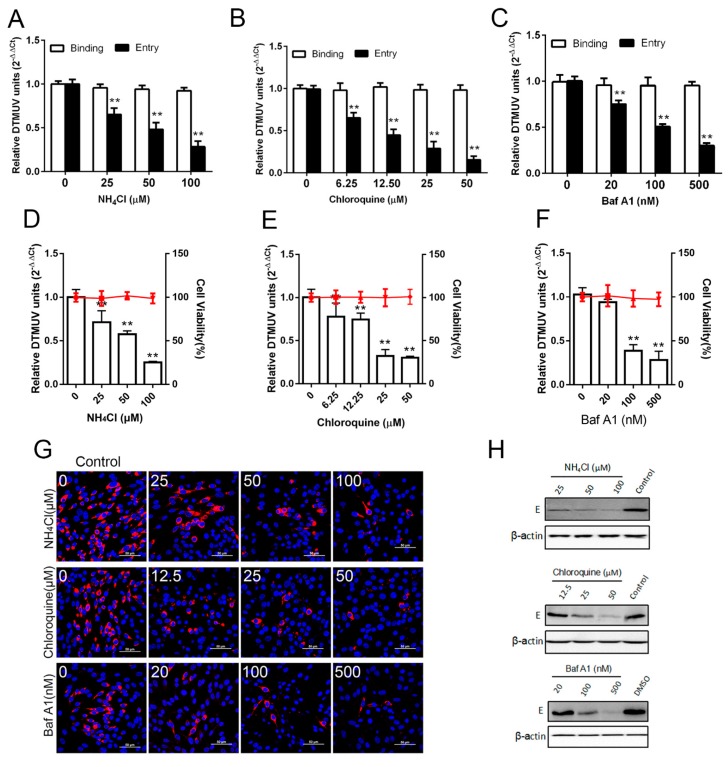
Acidic endosomal pH is required for entry and infection by Duck Tembusu virus (DTMUV). (**A**–**C**) DTMUV entry was inhibited by NH4Cl, chloroquine, and Bafilomycin A1 (Baf A1), but not binding. Subtoxic doses were used as a cell pretreatment at 37 °C for 1 h and 1 MOI of DTMUV was inoculated at 4 °C for 1 h. Following binding at 0 h or entry at 1 h post-infection at 37 °C, the amount of viral internalization was quantified by RT-qPCR after lysing the infected cells. (**D**–**F**) DTMUV infection was inhibited by chloroquine, NH4Cl, and Baf A1. Subtoxic doses were used as cells pretreatment at 37 °C for 1 h and 0.1 MOI of DTMUV was inoculated at 37 °C for 1 h. After 24 h post-infection (hpi), RT-qPCR was undertaken to check the viral load by lysing the infected cells. Subtoxic doses of chloroquine, NH4Cl, and Baf A1 on BHK-21 cells are shown in the horizontal lines, after being determined by cell viability assay. Means ± SDs of three independent trials are presented as results. **, *p* < 0.01. (**G**) Confocal microscopy showed the inhibition of the drugs. Different concentrations of NH4Cl, chloroquine, and Baf A1 were used as cell treatments for 1 h with infection with 0.1 MOI of DTMUV. At 24 hpi, monoclonal antibodies against DTMUV E were used for staining the cells after being fixed by 4% paraformaldehyde. DAPI was used for nuclei staining. Scale bar, 50 μM. (**H**) Western blotting showed the inhibition of the drugs. Cells were treated with NH4Cl, chloroquine, and Baf A1 with different concentrations for 1 h and infected with 0.1 MOI of DTMUV. At 24 hpi, Western blotting assay was performed with monoclonal antibodies against DTMUV E by lysing the cells.

**Figure 2:**
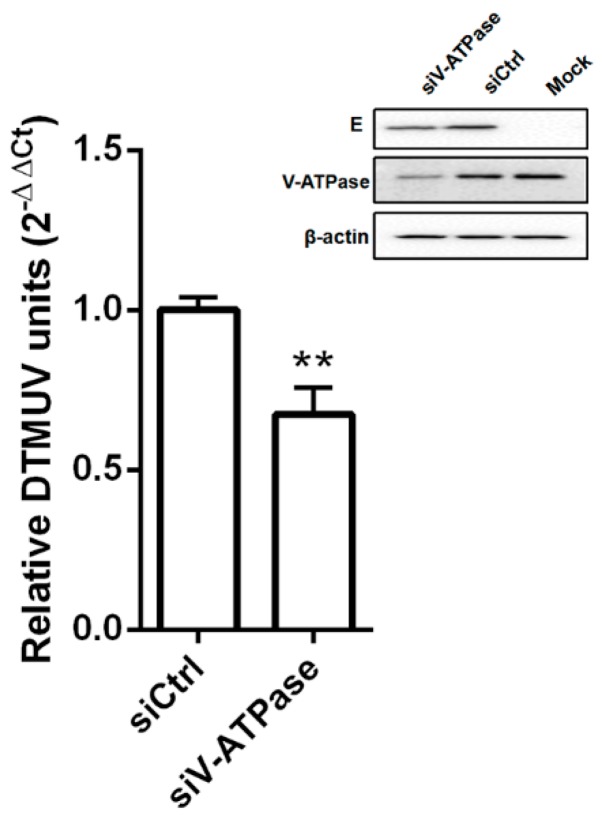
DTMUV infection was inhibited by V-ATPase knockdown. A 0.1 MOI of DTMUV was used for infection of siV-ATPase or siCtrl-transfected cells. At 24 hpi, RT-qPCR was performed to determine the viral load by lysing infected cells. Means ± SDs of three independent trials are presented as results. **, *p* < 0.01. Western blotting was done to check the V-ATPase knockdown effect on DTMUV infectivity. Anti-V-ATPase or anti-DTMUV E antibody was probed and expression of V-ATPase or DTMUV E was checked.

A number of viruses require exposure to an acidified environment for successful penetration and infection after internalization through endocytosis [21,22]. Treatment of cells with lysosomotropic agents, such as chloroquine and ammonium chloride, inhibits virus entry by preventing acidification [23]. In order to evaluate pH dependent DTMUV entry into cells, initially we studied the effects of two lysosomotropic agents (chloroquine and NH4Cl) and of Bafilomycin A1 (a potent inhibitor of V-ATPase and a particular inhibitor of the acidification of endosomal vesicles) on DTMUV infection in BHK-21 cells [24]. We found that chloroquine, NH4Cl, and Baf A1 inhibited DTMUV entry into cells but did not inhibit DTMUV binding to cells. The results also showed that the level of viral RNA was significantly decreased (Figure 1A–C). The concentrations of 50 μM chloroquine, 100 μM NH4Cl, and 500 nM Baf A1 caused 84%, 72%, and 70% reductions of the viral load, respectively. It was evident that the DTMUV entry process involves a low pH, and 24 hpi, RT-qPCR was performed to check the viral replication. All three inhibitors decreased DTMUV infection as compared with the control (Figure 1D–F). A concentration of 50 μM chloroquine, 100 μM NH4Cl, and 500 nM Baf A1 led to 72%, 75%, and 65% decreased amount of viral load, respectively. Subtoxic doses of the drugs were determined from cell proliferation assays (Figure 1D–F). Furthermore, confocal microscopy and Western blotting also showed that the DTMUV E proteins were decreased in a dose-dependent manner as compared with control (Figure 1G,H). Finally, we checked the requirement for a low pH for DTMUV entry by silencing the expression of vacuolar ATPase (V-ATPase), a proton pump key to establishing the low pH of the endosomal compartments [24]. Western blotting demonstrated that of small interfering RNA (siRNA) in the BHK-21 cells and treatment with siV-ATPase significantly decreased both the viral E protein level and the viral load (Figure 2). Altogether, the presented data demonstrated that entry of DTMUV into BHK-21 cells is dependent on pH.

### 3.2. Lysosomotropic Agents Act at an Early Time Point in DTMUV Infection

**Figure 3:**
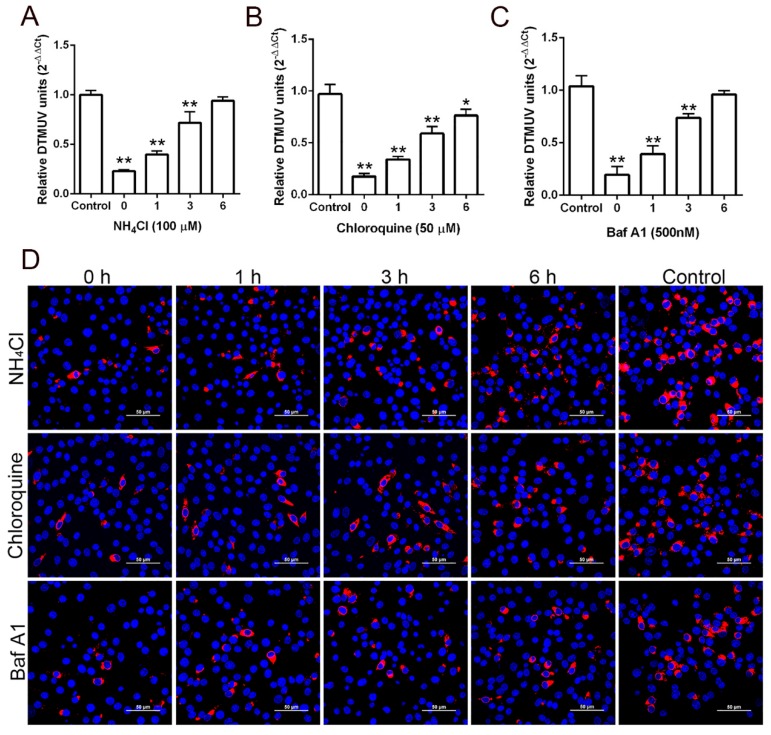
DTMUV entry effects by time course of lysosomotropic agents. DTMUV was bound to confluent cells in 24-well plates at 4 °C and remained there for 1 h. Sodium citrate buffer was used for inactivation of any non-internalized viruses, and NH4Cl (**A**), chloroquine (**B**), and Baf A1 (**C**) were added at 0–6 h post-infection. Infection continued for a total of 9 h. Sodium citrate buffer was used as a treatment for each time point for control wells, and untreated medium was added to the cells. RT-qPCR was undertaken to evaluate the viral replication by lysing the infected cells. Means ± SDs of three independent trials are presented as results. *, *p* < 0.05; **, *p* < 0.01. (**D**) BHK-21 cells were infected 1 h and NH4Cl, chloroquine and Baf A1 were added at 0–6 h. After 9 hpi the cells were fixed, permeabilized, and stained with DTMUV E antibody. DAPI was used for nuclei staining. Scale bar 50 μM.

A time of addition assay was performed to see whether lysosomotropic agents inhibited DTMUV at earlier or later time points of infection. BHK-21 cells were infected with DTMUV MOI 0.5 for 1 h at 37 °C. Then, the cells were replaced with fresh medium, NH4Cl, chloroquine, and Baf A1 (100 μM, 50 μM, and 500 nM, respectively) added at 0, 1, 3, and 6 h. After 9 hpi, the effects of the lysosomotropic agents were analyzed using RT-qPCR and confocal microscopy. Later treatment showed a lesser inhibitory effect on viral infection, which indicated that lysosomotropic agents are effective at an early stage of DTMUV infection (Figure 3). 

### 3.3. Effect of Moderate Acidic pH Pretreatment on DTMUV

**Figure 4:**
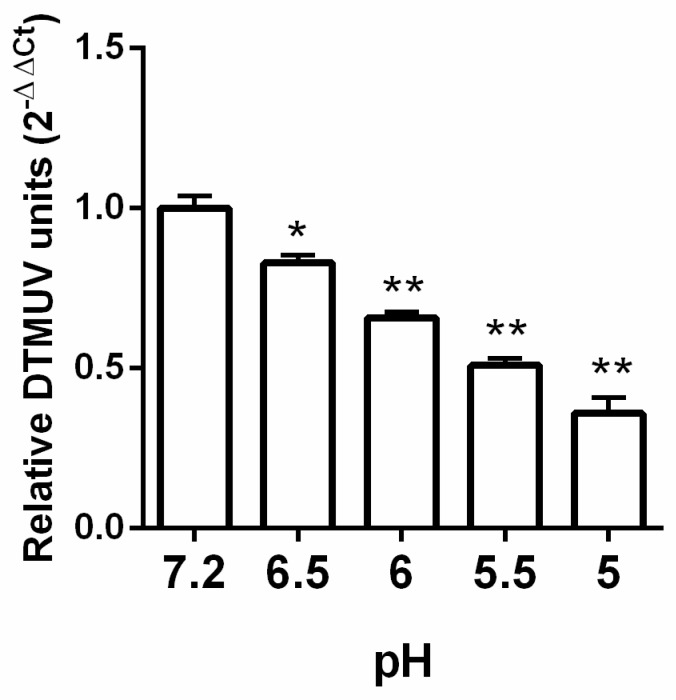
The moderate pH effect on DTMUV infection. DTMUV was incubated at different pH (7.2–5.0) at 37 °C for 30 min and pH 7.2 was set for neutralization before. The 24-well plates containing confluent cells were infected with treated virions for 6 h. RT-qPCR was undertaken to check the viral load by lysing the infected cells. Means ± SDs of three independent trials are presented as results. *, *p* < 0.05; **, *p* < 0.01.

Acidic pretreatment of virions that enter cells via a low-pH pathway inactivates their infectivity [25,26]. Exposure to a low-pH medium in the absence of a host target membrane is believed to impulsively prompt fusion glycoproteins of virions to change conformation in an irreversible manner, making the virus ineffective in fusion with host membranes [27]. Inactivation of DTMUV infectivity when exposed to a pretreatment of low pH was determined. Acidic conditions were provided to virions before infection of the cells. DTMUV virions were incubated at pH 7.2, 6.5, 6.0, 5.5, or 5.0 for 30 min; neutralized at pH 7.2; and then added to cells to check infectivity. Entry into BHK-21 cells was prevented by low-pH pretreatment, with a 65% reduction, compared with virions at pH 7.2 (Figure 4).

### 3.4. Effect of the Proteasome on DTMUV Entry

**Figure 5:**
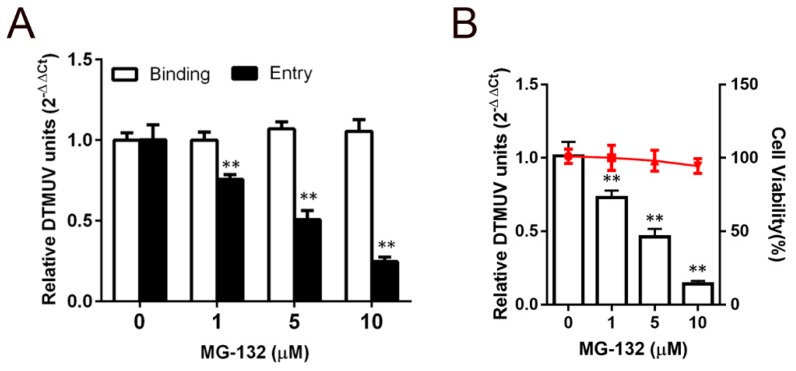
Effect of MG-132, a proteasome inhibitor, on DTMUV entry. (**A**) MG-132 inhibited DTMUV entry but not binding. Subtoxic doses were used as a cell pretreatment at 37 °C for 1 h and 1 MOI of DTMUV was inoculated at 4 °C for 1 h. Binding at 0 h or entry at 1 h at 37 °C were assessed by RT-qPCR to check the viral load by lysing the infected cells. (**B**) DTMUV infection was inhibited by MG-132. Subtoxic doses were used as a cell pretreatment at 37 °C for 1 h and 0.1 MOI of DTMUV was inoculated at 37 °C for 1 h. At 24 hpi, the amount of viral RNA was quantified by RT-qPCR. The cells are shown in horizontal lines, determined through the cell viability assay. Means ± SDs of three independent trials are presented as results. **, *p* < 0.01.

The eukaryotic proteasome system is a process in which the host cells degrade proteins and regulate cellular activities. Proteasome inhibition also decreases JEV entry into BHK-21 cells in vitro [28]. This suggests that a conserved proteasome-dependent mechanism can be employed by DTMUV, although the detailed mechanisms have yet to be elucidated. MG-132 is a peptide aldehyde that attaches to the active site of the 20S proteasome to act as a potent inhibitor of proteasome degradative activity. To define the role of the proteasome in DTMUV infection, we used MG-132 to figure out whether the proteasome was involved in DTMUV infection. Initially, we characterized the DTMUV life cycle during in the early events of binding and entry. BHK-21 cells were pretreated and inoculated with DTMUV MOI 1 at 4 °C and 37 °C for 1 h for binding and entry. Then the binding and entry events were quantified by RT-qPCR. The proteasome inhibitor MG-132 impaired entry without binding in a dose dependent manner (Figure 5A). Additionally, we measured MG-132 noncytotoxic concentrations using a cell viability assay (Figure 5B). The subconfluent BHK-21 cells were pretreated with the indicated concentrations of MG-132 for 30 min, and infected with DTMUV MOI 0.1 in the presence or absence of MG-132. After 6 hpi, multiplication of DTMUV was analyzed using RT-qPCR. The proteasome inhibitor MG-132 inhibited DTMUV multiplication in a dose dependent manner (Figure 5B). 

### 3.5. DTMUV Entry is Clathrin Dependent 

**Figure 6:**
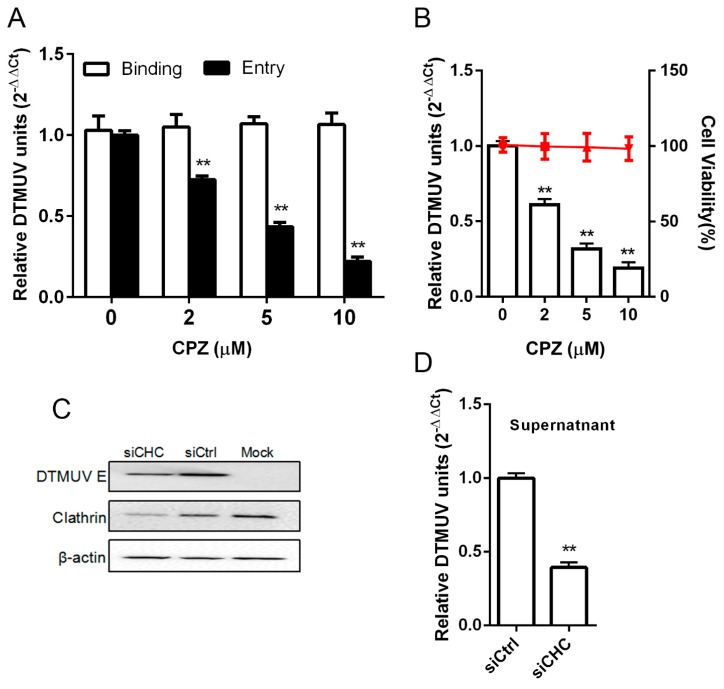
DTMUV entry is clathrin dependent. (**A**) DTMUV entry is inhibited by chlorpromazine (CPZ) but not binding. Subtoxic doses were used as a cell pretreatment at 37 °C for 1 h, the medium was replaced, and then 1 MOI of DTMUV was inoculated on the cells at 4 °C for 1 h. Binding at 0 h or entry at 1 h post-infection were assessed by RT-qPCR to check the viral load by lysing the infected cells. (**B**) DTMUV infection was inhibited by CPZ. An increased concentration of CPZ was used as a cell pretreatment for 1 h at 37 °C, the medium was replaced, and then 0.1 MOI of DTMUV was inoculated on the cells. At 24 hpi, RT-qPCR was undertaken to check the viral load by lysing the infected cells. The effects of subtoxic doses of CPZ on BHK-21 cells are shown in horizontal lines, determined through the cell viability assay. (**C**) Western blotting was used to check the clathrin heavy chain (CHC) knockdown effect on DTMUV infectivity. A 0.1 MOI of DTMUV was used for siCHC- or siCtrl-transfected cell infection. At 24 hpi, the expression of CHC and DTMUV E was probed with the indicated antibodies. (**D**) DTMUV infection was inhibited by clathrin knockdown, and 0.1 MOI of DTMUV was used for siCHC- or siCtrl-transfected cell infection. At 24 hpi, RT-qPCR was undertaken to check the viral load by lysing the supernatant. Means ± SDs of three independent trials are presented as results. **, *p* < 0.01.

Previous reports have suggested that calthrin-mediated endocytosis is used by JEV for entry into PK-15 or C6/36 cells [29,30]. Here, DTMUV entry into BHK-21 cells was determined by a series of experiments. Initially, cells were treated with different concentrations of chlorpromazine (CPZ), a potent inhibitor of clathrin. The results of CPZ treatment on DTMUV entry and infection were measured by RT-qPCR. We found that CPZ inhibited DTMUV entry but did not inhibit its binding (Figure 6A). Additionally, CPZ inhibited DTMUV infection (Figure 6B). The cell viability results showed no cytotoxic effect on cells (Figure 6B). The function of clathrin during DTMUV entry was further evaluated by siRNA knockdown of the clathrin heavy chain (CHC). A significant decrease of DTMUV E proteins and viral RNA was noticed in the siCHC-transfected cells, as compared to that in the siCtrl-transfected cells (Figure 6C). We concluded that DTMUV entry into BHK-21 cells was clathrin dependent.

## 4. Discussion 

Duck Tembusu virus (DTMUV) is a novel pathogenic member of the flaviviridae family and was first reported in South-Eastern China in 2010 in the coastal provinces [31]. The rapid spread of the disease can cause a serious decline in the egg production of laying ducks and neurological symptoms in ducks. Most *Flaviviruses* can cause zoonotic diseases and present different clinical symptoms, such as viral encephalitis and even systemic infection. Mosquito is the main host and principal vector of *Flaviviruses*. Under natural conditions, a variety of birds including ducks, chickens, and geese are at risk of DTMUV [8], affecting their growth and production [4]. DTMUV is an RNA virus with a single strand and positive-sense, which produces pathogenicity similar to that of other *Flaviviruses* [32]. It is well known that *Flaviviruses*, such as JEV, infect multiple cell types and have the capability to employ various endocytic pathways for entry into cells [33]. However, the endocytosis of DTMUV remains unclear. Therefore, this work aimed to investigate the mechanism of DTMUV entry. 

Host cell triggers such as low intracellular pH or receptor binding are often required to overcome the energy barrier for membrane fusion [25]. We evaluated here that DTMUV depends on low-pH exposure for entry into the BHK-21 cell line. In this study, chloroquine, NH4Cl, and Baf A1 inhibited 84%, 72%, and 70% of entry at the highest concentrations tested, respectively, suggesting that low-pH-mediated endocytosis was a leading route for DTMUV entry into BHK-21 cells. Our previous work showed that chloroquine, NH4Cl, and Baf A1 also inhibited the entry and replication of another important flavivirus, JEV [34]. The present study was in agreement with that early study. Furthermore, V-ATPase knockdown inhibited DTMUV infection. The results depicted here contribute to our understanding of DTMUV entry by explaining the role of endocytosis and exposure to low pH in BHK-21 cells. DTMUV is traverses a particular endocytic pathway, which is similar to most *Flaviviruses*. DTMUV, like many other *Flaviviruses* enters diverse pathophysiologically relevant cell types by employing distinct pathways. The DTMUV endocytic entry route is yet to be determined in primary cells. We suggest that an endosomal low pH is a significant trigger of fusion throughout DTMUV entry into certain cell types.

The host proteasome system 26s plays a vital role in the regulation and degradation of unwanted intracellular proteins in eukaryotic cells [35]. Previous studies have demonstrated that viral entry depends on the host proteasome system. Bovine Herpesvirus 1, Procine alpahaherpesvirus, JEV, and PCV2 entries are impeded by a proteasome inhibitor [25,28,36,37]. Here, we evaluated the role of the host proteasome in DTMUV entry. Pretreatment with MG-132, a peptide aldehyde inhibitor of the proteasome, did not affect the viral binding event but significantly decreased DTMUV entry by blocking the activity of the 26s proteasome. Our results revealed that the host proteasome system is necessary for the successful entry of DTMUV. 

*Flaviviruses* are globally important pathogens which affect humans and animals. These viruses enter through an endocytic process to infect a variety of cells. However, the mechanism of DTMUV internalization in BHK-21 cells has been unclear until now. Here, we identified DTMUV entry to be clathrin-dependent endocytosis. Chloropromazine is broadly used as an inhibitor of clathrin [38]. We observed that chloropromazine did not affect viral binding but DTMUV entry replication significantly decreased in a dose-dependent manner. Furthermore, a small interference of clathrin heavy chain (siCHC) knockdown decreased DTMUV replication, which is consistent with previous reports [34,39]. However, we suppose DTMUV can use the different pathways depending the host cell spectrum. In the future, we will determine whether this virus uses other endocytosis.

To our knowledge, this is the first report on DTMUV entry into BHK-21 cells via low endosomal pH and clathrin-mediated endocytosis. Our findings provide a novel insight into understanding the mechanism of DTMUV internalization and may be beneficial for the development of antiviral drugs that may be able to impede the early stages of DTMUV infection.
